# A Primary Hepatic Lymphoma Treated with Liver Resection and Chemotherapy

**DOI:** 10.1155/2014/749509

**Published:** 2014-08-04

**Authors:** Konstantinos Bouliaris, Grigorios Christodoulidis, Georgios Koukoulis, Ioannis Mamaloudis, Maria Ioannou, Eleni Bouronikou, Maria Palassopoulou, Konstantinos Tepetes

**Affiliations:** ^1^Surgical Department, University Hospital of Larissa, 41335 Mezourlo, Larissa, Thessaly, Greece; ^2^Pathology Department, University Hospital of Larissa, 41335 Mezourlo, Larissa, Thessaly, Greece; ^3^Hematology Department, University Hospital of Larissa, 41335 Mezourlo, Larissa, Thessaly, Greece

## Abstract

Primary hepatic lymphoma (PHL) is a rare malignancy, which is frequently misdiagnosed. Although chemotherapy is the treatment of choice there are reports that a combination of surgery and adjuvant chemotherapy can offer better results. Herein we present an interesting case of a large primary non-Hodgkin lymphoma originating from liver was treated with a liver which resection and chemotherapy.

## 1. Introduction

Primary hepatic lymphoma (PHL) is an extremely rare malignancy, accounting for less than 0.4% of extranodal non-Hodgkin lymphomas and 0.016% of all non-Hodgkin lymphomas [1]. The majority of PHL cases originate from B cells while T-cell lymphoma is less common. The etiology of PHL is unknown and although the liver contains lymphoid tissue, host factors seem to make the liver a poor environment for the development of malignant lymphomas [2]. Herein we present an interesting case of a large primary non-Hodgkin lymphoma originating from liver which was treated with a liver resection and chemotherapy.

## 2. Case Report 

**Figure 1:**
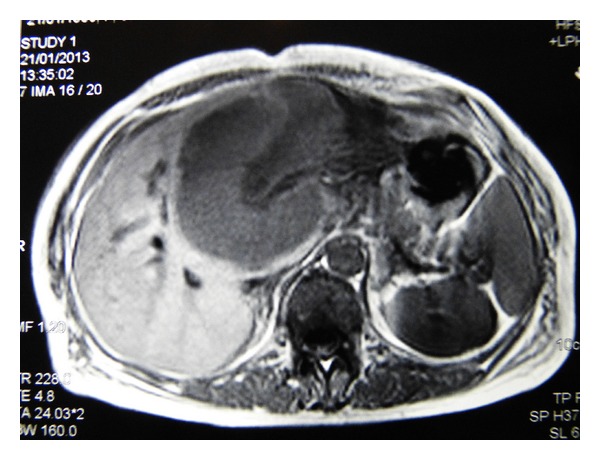
Axial T1-weighted MRI, showing a hypoattenuating hepatic lesion.

**Figure 2:**
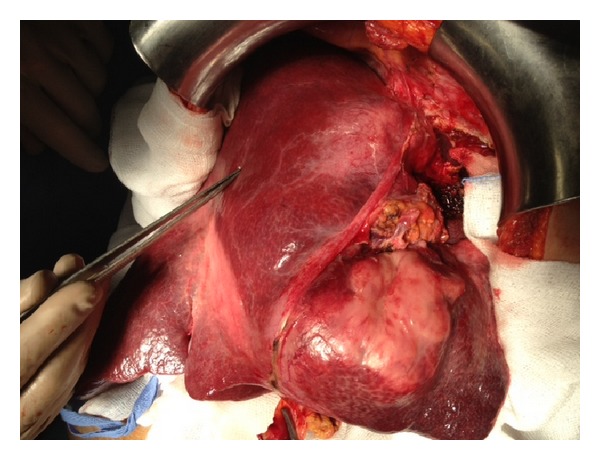
Intraoperative view of the lesion.

**Figure 3:**
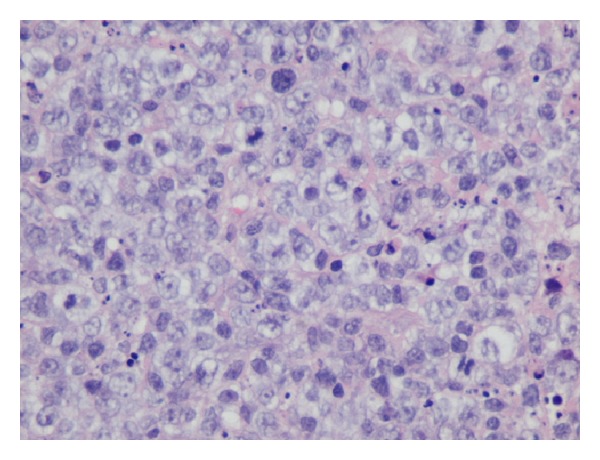
Infiltration of large lymphoid cells (haematoxylin and eosin stain, original magnification ×40).

**Figure 4:**
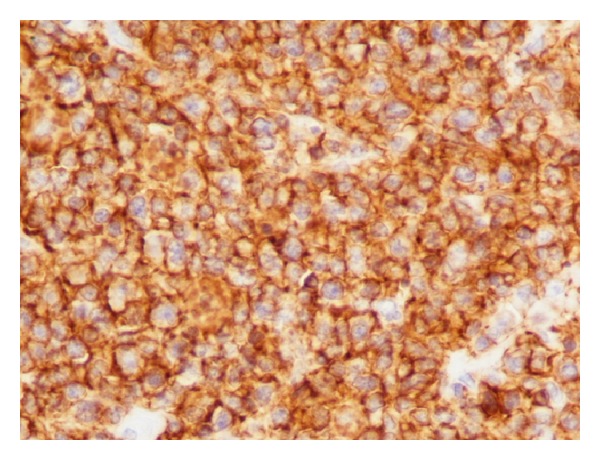
Positive CD20 (immunohistochemistry, original magnification ×40).

**Figure 5:**
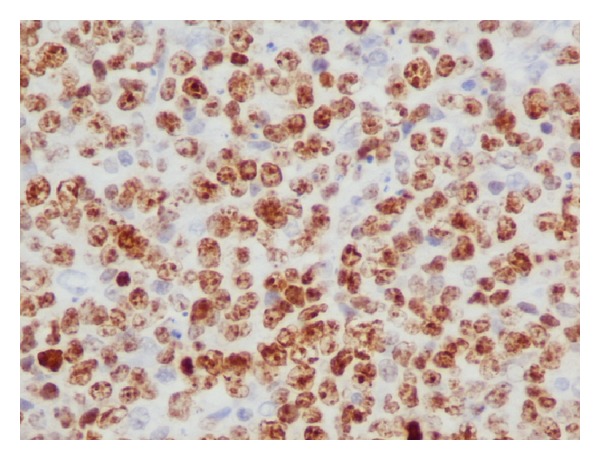
Positive Ki67 (immunohistochemistry, original magnification ×40).

A 57-year-old woman presented to the outpatient clinic complaining of upper abdominal discomfort, nausea, fatigue, intermittent low-grade fever, and weight loss in the last 5 months. Due to these symptoms a gastroscopy was done 4 months earlier which showed* H. pylori* infection for which she was given medical treatment. Otherwise, she had no remarkable past medical history. On physical examination a large, tender mass was found at the epigastrium with significant hepatomegaly but there was no splenomegaly or peripheral adenopathy. Peripheral blood cell counts revealed a mild anemia (hemoglobin 9.8 g/dL) and elevated white cell count (13.9 × 10^3^/*μ*L). Liver function tests were as follows: aspartate aminotransferase (AST) 47 IU (normal range <31); alanine aminotransferase (ALT) 22 IU (normal range <34); alkaline phosphatase (ALP) 134 IU (normal range <120); gamma-glutamyl transpeptidase (*γ*GT) 147 IU (normal range <38); and lactate dehydrogenase (LDH) 555 IU (normal range <247). Serological tests for hepatitis B and C virus were negative. Serology for HIV was negative too. The levels of tumors markers were cancer antigen 19-9 (Ca 19-9) 58.1 IU/mL (normal range <27), carcinoma antigen 15-3 (Ca 15-3) 31.5 IU/mL (normal range <5), and cancer antigen 125 (Ca 125) 88, 9 IU/mL (normal range <35). Alpha-fetoprotein (AFP) and carcinoembryonic antigen (CEA) were within normal values. Contrast-enhanced computed tomography (CT) revealed a large, solid mass (15 × 10 × 9 cm) occupying the left and partially the right hepatic lobe with heterogeneous enhancement. A magnetic resonance imaging (MRI) showed a low signal in T1 and high signal in T2 sequences mass with heterogeneous density (Figure 1). Our differential diagnosis included (1) primary hepatocellular cancer, (2) focal nodular hyperplasia, and (3) metastasis from gastrointestinal cancer. The latter was ruled out with a gastroscopy and a colonoscopy. The tumor was symptomatic and potentially respectable and thus an exploratory laparotomy was carried out. During the operation a solid and well-circumscribed mass was found on the left and partially on the right hepatic lobe and an extended left hepatectomy was performed (Figure 2). No palpable lymph nodes were evident in the mesentery, the hepatoduodenal ligament, or the retroperitoneal space. The gastrointestinal tract was normal too. The histopathological examination showed large lymphoid cells with oval to round vesicular nuclei containing fine chromatin. There were one, two, or multiple nucleoli and numerous mitotic figures. Necrotic cells were also observed (Figure 3). Immunohistochemistry was positive for the pan-B-cell marker CD20 (Figure 4), as well as LCA and CD43, while it was negative for CD3, CD5, CD10, CD138, MUM1, bcl6, and bcl2. The lymphoid cells showed also a high proliferation fraction as detected by Ki67 (MIB-1) immunostaining (Figure 5). The diagnosis of B-cell non-Hodgkin lymphoma of high malignancy was made. The patient had an uneventful recovery with regression of B symptoms and she was discharged on the 10th postoperative day. She was referred to the department of hematology for further treatment. Bone marrow aspiration as well as thoracic and neck CT scans was normal. The patient was given four cycles of chemotherapy including rituximab, cyclophosphamide, hydroxydaunorubicin, Oncovin, and prednisone (R-CHOP) and twelve months after the operation she is disease-free.

## 3. Discussion

Primary hepatic lymphoma (PHL) is defined according to Caccamo's criteria as lymphoma confined only to the liver without the involvement of any other organ like spleen, bone marrow, lymph nodes, peripheral blood, or other tissues until at least six months after diagnosis [3]. It can occur at any age but is usually found in the fifth or sixth decade of life, with male/female ratio of 2-3/1 [4]. The pathogenesis of PHL is still unclear and it has been associated with EBV, HCV, HIV, or HTLV infections, liver cirrhosis, systemic lupus erythematosus, and immunosuppressive therapy [5]. However, our patient had none of the above conditions or risk factors for PHL. The clinical manifestations are atypical including abdominal pain, fever, weight loss, and night sweats.

Hepatomegaly is the most frequent finding. Liver function tests are usually abnormal with elevation of ALP and LDH. The latter is increased in 30–80% of the cases [5]. On the contrary, tumor markers are usually within normal ranges with aFP and CEA being normal in almost 100% of the patients. Elevated LDH, with normal AFP and CEA, constitutes a valuable biologic feature [6] but it was not evaluated in our case. Radiological features of PHL are usually nonspecific and the most common presentation on CT scan is a solitary hypoattenuating lesion, which may have a central area of low intensity indicating necrosis [5]. Findings on MRI are variable too, with the most common being hypointense T1-weighted and hyperintense T2-weighted lesions [7]. Due to the rarity of this disease entity and the nonspecific clinical presentation, laboratory, and radiologic features, PHL may be confused with hepatitis, primary hepatic tumors, hepatic metastases, and systemic lymphoma with secondary hepatic involvement. Diagnosis of PHL requires a liver biopsy compatible with lymphoma and the absence of lymphoproliferative disease outside the liver. In our case liver biopsy was not performed for the following reasons: (1) there were no laboratory features suggestive of PHL; (2) the lesion was profoundly symptomatic; and (3) it was considered and proved to be resectable. In fact it was considered as alpha-fetoprotein-negative hepatocellular carcinoma, possibly of the fibrolamellar pattern.

The prognosis of PHL is poor with a median survival time of 15 months. However, it varies widely, ranging from 3 to 124 months [8]. Treatment options for PHL include surgery, chemotherapy, radiation, or varying combinations of these modalities [5, 8]. Although the optimal treatment is not yet defined, chemotherapy with CHOP-based regimens is the gold standard [5, 9]. The role of surgery is not fully clarified but there are reports that liver resection followed by adjuvant chemotherapy and/or radiation results in better prognosis [5, 10, 11]. Yang et al. in a small retrospective analysis of nine patients with PHL treated with liver resection showed that surgery followed by chemotherapy had a better outcome and that postoperative chemotherapy was the only prognostic factor for survival. They conclude that good prognosis of PHL can be obtained by early surgery combined with chemotherapy in strictly selected patients [12]. Similarly, Avlonitis and Linos in a large review of the literature showed that patients treated with surgery followed by chemotherapy had better survival rates [8]. Extrapolating from the available data, the current indications for surgery include patients with localized disease that can undergo R0 resection or cases in which there is persistent respectable disease following chemotherapy.

## 4. Conclusions 

Primary hepatic lymphoma is a rare malignancy, which is frequently misdiagnosed. However, it should be considered in cases of space occupying liver lesions with elevated LDH and normal AFP and CEA. If the index of suspicion for PHL is high, a liver biopsy should be obtained. PHL is chemosensitive and early aggressive combination of chemotherapy may result in sustained remission. The role of surgery has not yet been clarified but, in patients with localized disease, surgery followed by adjuvant chemotherapy should be considered to prevent recurrence.
